# *Drosophila melanogaster* as a Model System to Assess the Effect of Epstein-Barr Virus DNA on Inflammatory Gut Diseases

**DOI:** 10.3389/fimmu.2021.586930

**Published:** 2021-03-22

**Authors:** Joelle R. Madi, Amani Al Outa, Mirna Ghannam, Hadi M. Hussein, Marwa Shehab, Zeinab Al Kobra Haj Hasan, Antoine Abou Fayad, Margret Shirinian, Elias A. Rahal

**Affiliations:** ^1^ Department of Experimental Pathology and Immunology, American University of Beirut, Faculty of Medicine, Beirut, Lebanon; ^2^ Center for Infectious Diseases Research, American University of Beirut Medical Center, Beirut, Lebanon; ^3^ Department of Anatomy, Cell Biology and Physiology, American University of Beirut, Faculty of Medicine, Beirut, Lebanon

**Keywords:** epstein-barr virus, inflammation, gut, hemocytes, DNA

## Abstract

The Epstein-Barr virus (EBV) commonly infects humans and is highly associated with different types of cancers and autoimmune diseases. EBV has also been detected in inflamed gastrointestinal mucosa of patients suffering from prolonged inflammation of the digestive tract such as inflammatory bowel disease (IBD) with no clear role identified yet for EBV in the pathology of such diseases. Since we have previously reported immune-stimulating capabilities of EBV DNA in various models, in this study we investigated whether EBV DNA may play a role in exacerbating intestinal inflammation through innate immune and regeneration responses using the *Drosophila melanogaster* model. We have generated inflamed gastrointestinal tracts in adult fruit flies through the administration of dextran sodium sulfate (DSS), a sulfated polysaccharide that causes human ulcerative colitis- like pathologies due to its toxicity to intestinal cells. Intestinal damage induced by inflammation recruited plasmatocytes to the ileum in fly hindguts. EBV DNA aggravated inflammation by enhancing the immune deficiency (IMD) pathway as well as further increasing the cellular inflammatory responses manifested upon the administration of DSS. The study at hand proposes a possible immunostimulatory role of the viral DNA exerted specifically in the fly hindgut hence further developing our understanding of immune responses mounted against EBV DNA in the latter intestinal segment of the *D. melanogaster* gut. These findings suggest that EBV DNA may perpetuate proinflammatory processes initiated in an inflamed digestive system. Our findings indicate that *D. melanogaster* can serve as a model to further understand EBV-associated gastroinflammatory pathologies. Further studies employing mammalian models may validate the immunogenicity of EBV DNA in an IBD context and its role in exacerbating the disease through inflammatory mediators.

## Introduction

The Epstein-Barr virus (EBV), also referred to as the *Human herpesvirus 4* (HHV-4), belongs to the *Herpesvirinae* family and is capable of establishing life-long latency in the infected host. EBV has been associated with several types of malignancies; infection with the virus has been shown to downregulate tumor suppressor and DNA repair proteins (1) some of which are required for maintaining genomic stability (2). EBV has been reported to frequently reactivate in the infected host thus resulting in the consistent shedding of viral antigens, such as DNA, that may trigger immune responses. The EBV genome consists of linear, double stranded DNA that is approximately 172 kilobase pairs in length and that encodes for more than 85 genes (3). EBV DNA was shown to have immunomodulatory properties in multiple systems (4). It was reported to trigger interferon alpha (IFN-∝) and IL-8 from human monocytes and plasmacytoid dendritic cells in a Toll-like receptor 9 (TLR9)-dependent manner. TLR9 is an endosomal pattern recognition receptor (PRR) known to recognize CpG (cytosine-phosphodiester bond-guanine)-rich unmethylated DNA such as that of the nascent EBV genome (5).We further showed that EBV DNA has immune stimulatory capabilities in mice *via* enhancing the production of the pro-inflammatory interleukin 17A (IL-17A), a cytokine associated with autoimmune diseases such as rheumatoid arthritis (RA), multiple sclerosis (MS) and systemic lupus erythematosus (SLE). This coincided with an increase in IL-23, another pro-autoimmune cytokine required for sustaining the IL-17A response (6). Furthermore, EBV DNA was capable of modulating IL-17A *via* TLR3, 7 and 9 in mice and peripheral blood mononuclear cells (PBMCs) (7). Additionally, we identified a linear correlation between EBV DNA copy numbers and IL-17A levels in RA patients, which was not observed in non-RA controls (8). In a recent study using the invertebrate *Drosophila melanogaster* model, we showed that EBV DNA stimulates an increase in circulating hemocyte numbers through triggering the immune deficiency (IMD) pathway, which is comparable to the tumor necrosis factor receptor (TNFR) signaling pathway in mammals. Expression of the pro-inflammatory cytokine TNF-∝ was also increased in mice injected with EBV DNA (9). EBV is associated with infectious mononucleosis in addition to various types of cancers and immune-mediated diseases such as inflammatory bowel disease (IBD). IBD includes Crohn’s disease and ulcerative colitis, conditions that are associated with chronic inflammation of the gut. Several factors highlight the need to evaluate the role played by EBV in inflammatory diseases of the gastrointestinal tract. EBV is present at low levels in healthy gastric and colonic mucosa but has been detected at higher levels in various types of inflammatory gastrointestinal conditions including gastritis lesions, Crohn’s disease and ulcerative colitis (10–14). The role EBV plays in these conditions is still unknown. Based on our previous findings indicating a proinflammatory effect for EBV DNA, genetic material from this virus may play similar roles in IBD. Hence, we used *D. melanogaster* as a simple but efficient system (15) that allows modeling of human intestinal diseases; this is specifically due to the presence of a high degree of conservation between *D. melanogaster* and mammalian intestinal anatomy, histology, development, regeneration and the signaling pathways that control them (16). Hence, in the current study we aimed to identify whether EBV DNA plays a role in gut pathology focusing specifically on the innate immune system and gut regeneration processes.

## Materials and Methods

### Fly Stocks and Crosses

Flies were maintained on regular fly food at 25°C. Standard *Drosophila* husbandry procedures were used at 25°C). The *Hml-Gal4,UAS-GFP* (Bloomington *Drosophila* Stock Center: 30139, a gift from Dan Hultmark), *esg-Gal4*, *UAS-GFP* (Bloomington *Drosophila* Stock Center: 67054) and wild type *W^1118^* flies (Bloomington *Drosophila* Stock Center: 3605) were used in this study. The *Hml-Gal4,UAS-GFP* line carries a 3.0 kb region upstream of the hemolectin gene fused to the coding sequence of Gal4 along with UAS-GFP; this allows production of GFP in hemolectin-expressing cells (17). On the other hand, the *esg-Gal4*, *UAS-GFP* line harbors the P{GawB} P-element construct; this construct carries Scer\GAL4 driver/enhancer trap sequences and similarly allows for GFP production in escargot expressing cells (18). Newly hatched day 0 males were collected and employed in the experimental procedures described below. The *Hml-Gal4,UAS-GFP.*

### Treatments

**Figure 1:**
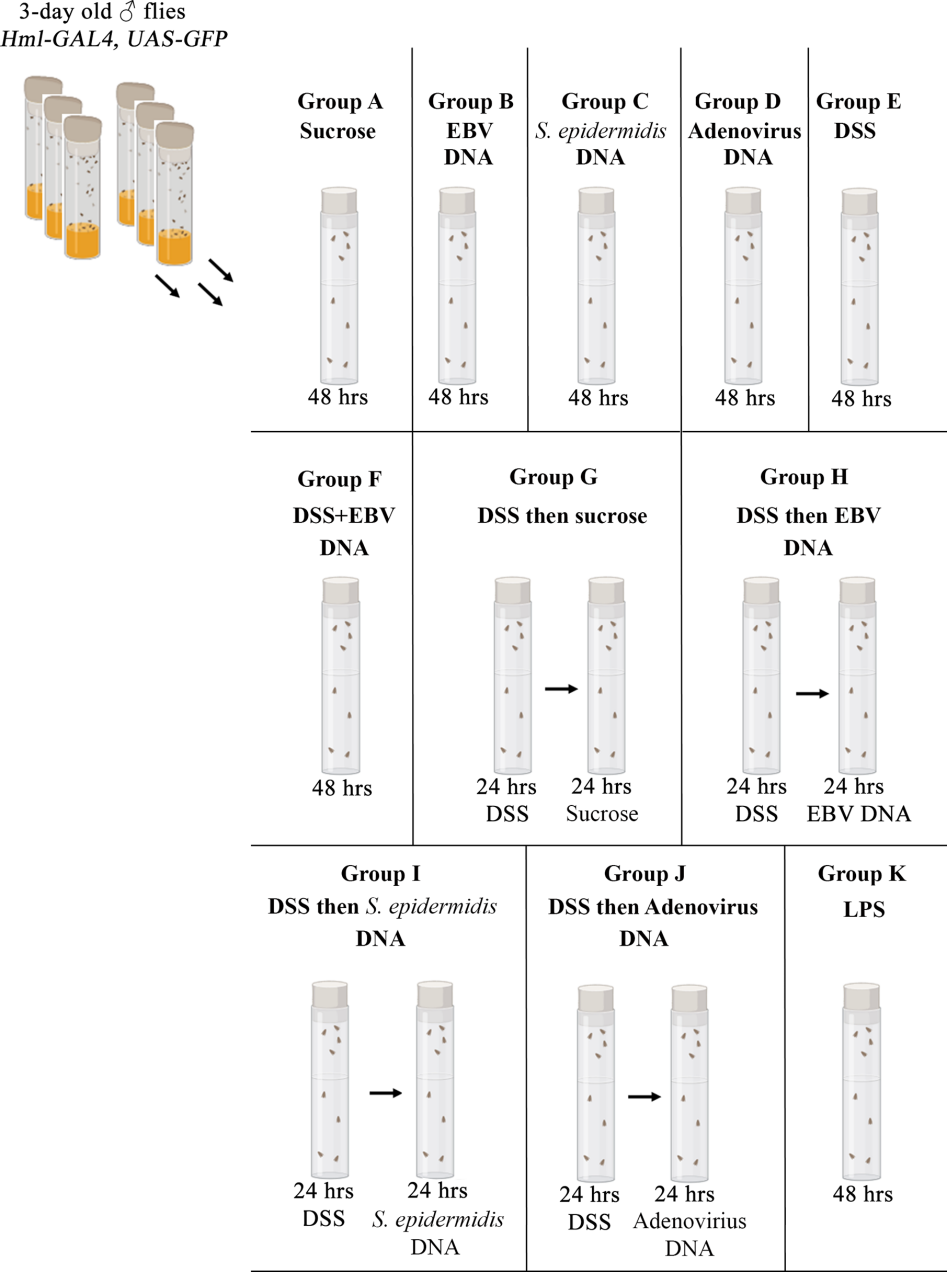
Feeding regimen. Three-day old male flies were fed on different regimens of sucrose, DSS, EBV DNA, *S. epidermidis DNA*, Adenovirus DNA or LPS for 48 hours as indicated. Vials were fitted with a filter paper onto which the various treatments were applied.

Three-day old adult male flies were transferred from feeding on regular fly food onto a filter paper (Bio-rad, Hercules, CA) fitted at the base of a vial. All agents described below were dissolved in 5% sucrose and applied onto this filter paper in volumes of 500 µl. Treatments consisted of sucrose alone, 5% DSS (Abcam, Cambridge, UK), 288,000 copies of EBV DNA (Vircell Microbiologists, Granada, Spain), 44.6 pg of *Staphylococcus epidermidis* DNA (equivalent to the weight of 144,000 EBV DNA copies), 288,000 copies of Adenovirus DNA (Vircell Microbiologists) or 0.5 mg of lipopolysaccharide (LPS) from *Escherichia coli* O111:B4 (InvivoGen, Toulouse, France). These treatments were given alone or in succession as indicated in **Figure 1**. The *S. epidermidis* and Adenovirus DNA were used as bacterial and viral control DNA, respectively, while the LPS was used as a control for inflammation induction. To verify that flies were feeding off the filter paper, wild type flies were fed on fluorescein-labeled EBV DNA (Advanced Biotechnologies, Colombia, MD) or methylene blue to monitor the uptake and passage of ingested elements. All groups were fed at 25°C before gut dissections were performed.

### EBV DNA Fluorescent Labeling

EBV DNA was labeled using NHS-fluorescein (Abcam), 1.5 equivalence, dissolved in 100 µl of nuclease-free water. One ml of sodium hydrogen carbonate (1 M, pH 8-9) and 10 µl of purified EBV DNA (24,000 copies/µl) were added to the prepared NHS-fluorescein. The mixture was incubated overnight at room temperature on a shaker at low speed. Then, the labeled EBV DNA was purified from the mixture using reverse-phase chromatography. C18 columns with inert glass, reverse-phase silica and a gradient of water and methanol was used. The water-methanol ratio increased gradually by 10% of methanol every 3 mL of elution. The reaction mixture was loaded into the columns then 50 µl of the purified mixture was obtained from the column every 10 minutes over a period of 5 hours. The 50 µl aliquots were lyophilized and then stored at -20°C. To assess the purity of the labeled DNA, thin layer chromatography (TLC) was performed on the DNA dissolved in nuclease-free water using silica-coated TLC Aluminium Sheets (Sigma-Aldrich, Saint Louis, MO). The mobile phase consisted of 5 ml of 100% ethyl acetate. Visualization was performed using UV light at 256 nm and 360 nm.

### Dissections, Immunostaining and Fluorescent Microscopy

Three-day old *Hml-Gal4, UAS-GFP* and *esg-Gal4*, *UAS-GFP/Cyo* flies subjected to treatments as described above were dissected. Whole guts were pulled from the posterior end of the flies using forceps (Electron Microscopy Sciences, Dumont, Number 5, Hatfield, PA) directly into 1X phosphate-buffered saline (PBS) on a cold glass plate. Guts were then fixed in 4% formaldehyde (Sigma-Aldrich) for 20 minutes followed by three washes of 10 minutes each using 1X PBS- 0.3% Triton X-100 (PBST). For immunostaining, guts from *Hml-Gal4, UAS-GFP* were placed in blocking solution (5% normal goat serum (Dako, Santa Clara, CA) for 30 minutes and subsequently incubated overnight with a mixture of primary antibodies diluted in blocking solution; the mouse anti-P1 IgG (Kind gift from Istvan Ando, 1:100) and rabbit anti-Green Fluorescent Protein (GFP) IgG (Abcam, 1:500) primary antibodies were used. Guts were then washed in PBST three times for 10 minutes and incubated with the AlexaFluor-594 anti-mouse IgG (Abcam, 1:500) and AlexaFluor-488 anti-rabbit IgG (Abcam, 1:500) secondary antobodies (19). After two hours of incubation with the secondary antibodies, guts were washed with PBST three times for 10 minutes. All samples, including those that were not immunostained, were mounted onto microscope slides using Fluoroshield Mounting Medium with DAPI (Abcam). The entire dissection/fixation process was performed on ice. Images were acquired using a laser scanning confocal microscope (Carl Zeiss Laser Scanning Microscopy 710, Jena, Germany). For experiments verifying that adult wild type flies fed on DNA as indicated above, images were taken on a fluorescent stereomicroscope (Olympus SZX 10, Waltham, MA).

### Hemocyte Counts in Adult Fly Guts

Hemolectin-positive GFP cells in guts obtained from flies treated as described above (~50 guts per group) was performed using the *Hml-Gal4, UAS-GFP* fly line, the Image-based Tool for Counting Nuclei (ITCN) plugin on ImageJ 1.49v was used. The following parameters were employed: a width of 10 pixels, a minimum distance of 10 pixels and a threshold of 0.8 without detecting any dark peaks. Statistical significance was analyzed using the chi squared test. P-values less than 0.05 were considered statistically significant.

### Circulating Hemocyte Counts in Adult Flies

Five *Hml-Gal4, UAS-GFP* adult flies per group were bled in 20µl of 1X PBS on a Parafilm strip by pricking the flies on their thorax using a needle. The adult flies were allowed to bleed for 30 seconds before 10 µl of the bleed was removed and examined using a hemocytometer under a light microscope employing a 40X magnification. Hemocyte counts were performed in triplicates for each group studied. To analyze statistical significance, the unpaired t-test was performed. P-values less than 0.05 were considered statistically significant.

### Midgut Intestinal Stem Cell Regeneration Analysis

To analyze midgut intestinal stem cell (ISC) regeneration, the fly line *esg-Gal4*, *UAS-GFP* was used. The cells marked by *esg* are restricted to the midgut region. The fluorescence intensity measure in the midgut of each group was assessed using ImageJ 1.49v. The unpaired t-test was then conducted to assess statistical significance. P-values less than 0.05 were considered significant.

### RNA Extraction

Three-day old *Hml-Gal4, UAS-GFP* flies were treated as detailed above; their guts were then dissected and RNA was extracted for real time PCR analysis (~60 guts per group). Two different RNA extraction protocols were followed depending on the sample type: RNA extraction using TRI reagent (Sigma-Aldrich) was employed for all fly groups that were not fed on DSS following manufacturer recommendations (20). DSS causes DNA polymerase and reverse transcriptase inhibition hence intervening with real time PCR assessment unless removed (21). Therefore, for DSS-fed fly groups, RNA extraction was performed using TRI reagent and lithium chloride (Sigma-Aldrich). A volume of 300 µl of TRI reagent was added onto 60 guts from each group fed on DSS. The guts were homogenized using a pestle and incubated for 5 minutes at room temperature. Then, 288 µl of chloroform were added and tubes were shaken vigorously for 15 seconds. Tubes were left at room temperature for 3 minutes before 132 µl of isopropanol were added. Tubes were shaken vigorously again for 15 seconds before centrifugation at 12,000 g for 10 minutes at 4°C. Supernatants were then transferred into a new tube containing lithium chloride with a final concentration of 2 M and incubated overnight at 4°C. Subsequently, tubes were centrifuged at 12,000 g for 10 minutes at room temperature. The supernatant was discarded and the pellet was washed with an equal amount of 2 M lithium chloride. The sample was centrifuged at 12,000 g for 10 minutes at 4°C. The supernatant was discarded again and the pellet was washed twice with 300 µl of 70% ethanol; each wash was followed by centrifugation at 12,000 g for 10 minutes at 4°C. The pellet was then resuspended in 10 µl RNase-free water (22). The entire procedure was completed on ice. The concentration and purity of the RNA were measured using a microspectrophotometer (Denovix Tc 312, Wilmington, DE).

### Relative Gene Expression Analysis

To assess the relative expression of *Diptericin* and IMD in three-day old *Hml-Gal4, UAS-GFP* flies after being treated as described above, real time PCR was conducted (23–26). cDNA synthesis was performed using the iScript cDNA Synthesis Kit (Bio-rad) according to the manufacturer’s specifications. Briefly, reaction tubes included 1 µl Reverse Transcriptase, 4 µl Reaction Mix and 500 ng of RNA in 20 µl reactions. Samples were then incubated in a thermal cycler (Thermo Electron Corporation, Waltham, MA) undergoing the following steps: priming for 5 minutes at 25°C, reverse transcription for 20 minutes at 46°C, reverse transcription inactivation for 1 minute at 95°C and a hold step at 4°C. The final cDNA products were stored at -20°C for later use (27, 28). *Ribosomal protein L 11* (*RPL 11*) was used as a housekeeping gene. For real time analysis, PCR reactions consisted of 5 µl SYBR (Bio-Rad) green, 1µl of the forward primer at a concentration of 10 pmol/uL, 1µl of the reverse primer at a concentration of 10 pmol/uL and 300 ng of cDNA in a total volume of 10 µl per sample. Each sample was run in triplicates using the following program: initial activation step at 95°C for 5 minutes followed by 40 cycles consisting of 95°C for 15 seconds and 57°C for 30 seconds. Primers were obtained from Macrogen (Seoul, South Korea). The forward and reverse primers for *Diptericin* had the following sequences: 5’-AAGTGGGAAGCACCTACACCTACA-3’ and 5’-GTATCTTCCGGACAGGCAGT-3’; those for IMD had the sequences 5’-TCAGCGACCCAAACTACAATTC-3’ and 5’-TTGTCTGGACGTTACTGAGAGT-3’ while those for RPL11 had the sequences 5’-CGATCCCTCCATCGGTATCT-3’ and 5’-AACCACTTCATGGCATCCTC-3’ (29). Real time detection was performed using the Bio-Rad CFX96 Real Time System. Relative gene expression analysis was determined using the Livak method. Assessments were performed in triplicates. To analyze statistical significance, the unpaired t-test was performed. P-values less than 0.05 were considered statistically significant.

## Results

### Increased Hindgut Hemocyte Numbers in Flies Fed on DSS then EBV DNA

**Figure 2:**
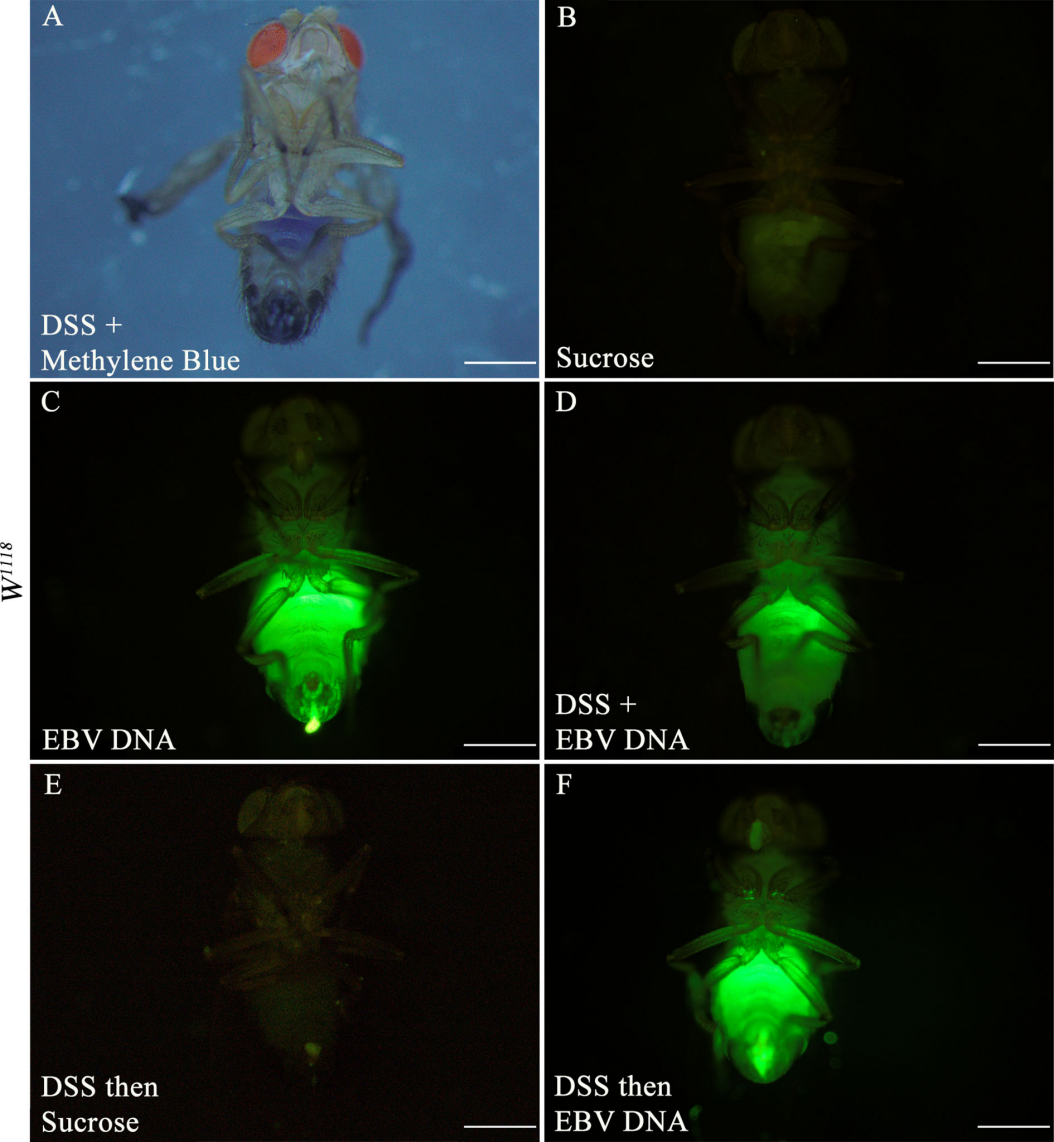
Three-day old *W^1118^* males fed on different regimens of DSS and/or fluorescein-labeled EBV DNA for 48 hours. **(A)** *W^1118^* fly fed on DSS and methylene blue. **(B)** *W^1118^* fly fed on sucrose (used as vehicle in the other treatments) for 48 hours. **(C)** *W^1118^* fly fed on fluorescein-labeled EBV DNA for 48 hours. **(D)** *W^1118^* fly fed on DSS and fluorescein-labeled EBV DNA for 48 hours. **(E)** *W^1118^* fly fed on DSS for 24 hours then sucrose for 24 hours. **(F)** *W^1118^* fly fed on DSS for 24 hours then on fluorescein-labeled EBV DNA for 24 hours. Scale bar indicates 500 μm.

To examine the effect of EBV DNA on the *D. melanogaster* gut, flies were fed various regimens that included EBV DNA **(** **Figure 1** **).** To validate flies fed on such regimens, fluorescein-labeled EBV DNA was added to either sucrose or DSS and then applied to a filter paper. Twenty wild type flies were used per feeding regimen and after a period of 48 hours of feeding, fluorescence in the abdominal and proboscis regions of the flies was observed (**Figure 2**). All flies that were fed EBV DNA or DSS with EBV DNA (**Figures 2C, D, F**) showed fluorescence hence confirming ingestion of the EBV DNA even in the presence of DSS.

**Figure 3:**
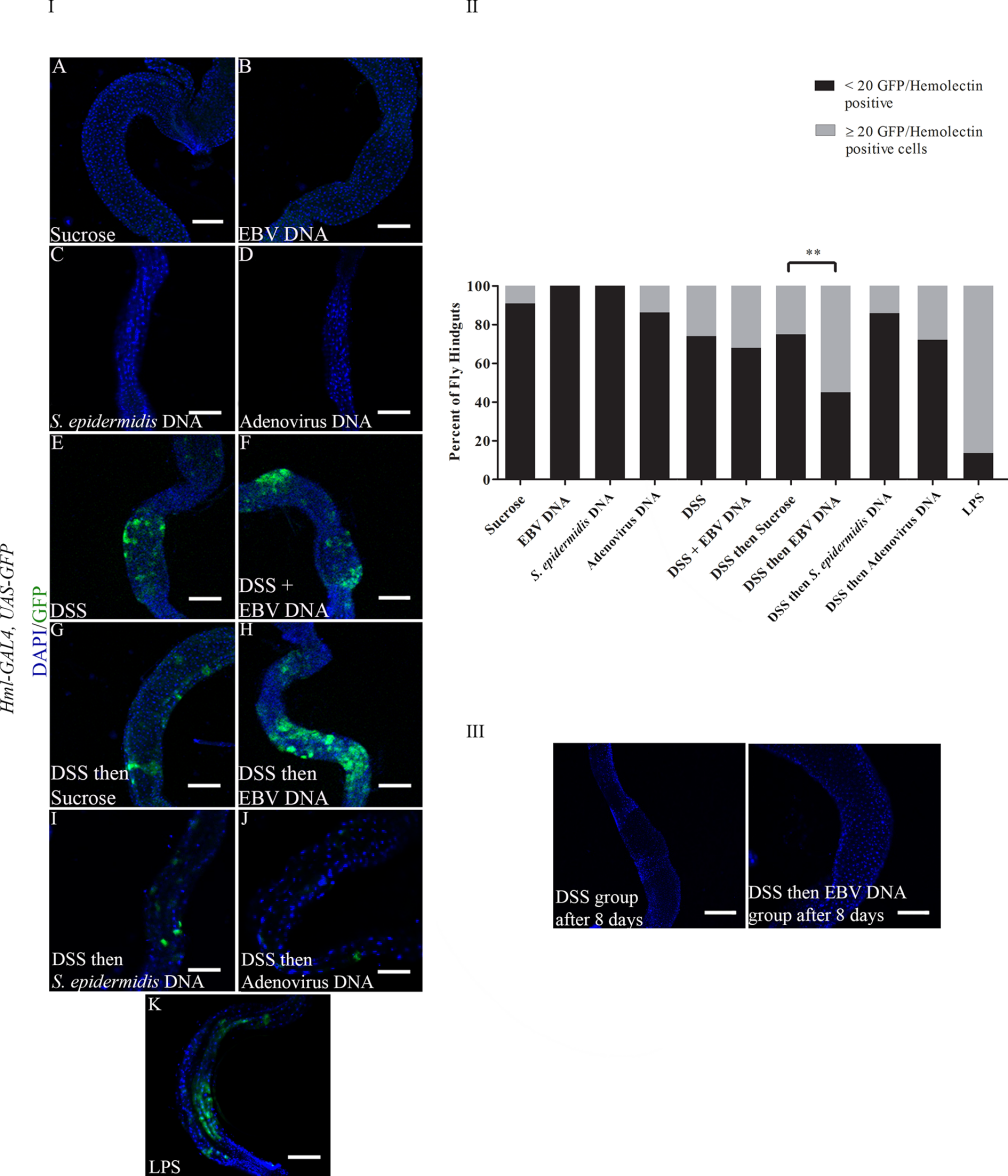
(I): Hindguts of *Hml-Gal4, UAS-GFP* flies fed on different regimens of DSS, EBV DNA, *S. epidermidis* DNA, Adenovirus DNA or LPS **(A)** Flies fed on sucrose (used as vehicle in the other treatments) for 48 hours. **(B)** Flies fed on EBV DNA for 48 hours. **(C)** Flies fed on *S. epidermidis* DNA for 48 hours. **(D)** Flies fed on Adenovirus DNA for 48 hours. **(E)** Flies fed on DSS for 48 hours. **(F)** Flies fed on DSS and EBV DNA for 48 hours. **(G)** Flies fed on DSS for 24 hours then sucrose for 24 hours. **(H)** Flies fed on DSS for 24 hours then EBV DNA for 24 hours. **(I)** Flies fed on DSS for 24 hours then *S. epidermidis* DNA for 24 hours. **(J)** Flies fed on DSS for 24 hours then Adenovirus DNA for 24 hours. **(K)** Flies fed on LPS for 48 hours. GFP-Hemolectin is in green; DAPI is in blue. Scale bar indicates 100 μm. (II): Percent of fly hindguts with GFP-Hemolectin positive cells after feeding on different treatments of DSS, EBV DNA, *S. epidermidis* DNA, Adenovirus DNA or LPS. Treatments were administered as indicated in Panel (I) Sucrose was used as vehicle. **p-value<0.01 compared to flies fed on DSS for 24 hours then sucrose for 24 hours. (III): Hindguts of *Hml-Gal4, UAS-GFP* flies fed on DSS for 24 hours then sucrose for 24 hours or DSS for 24 hours then EBV DNA for 24 hours. Hindguts are depicted after 8 days of treatment cessation.

To examine whether DSS and EBV DNA treatments affect the number of hemocytes in the fly gut, the *Hml-Gal4,UAS-GFP* fly line was used. Hemolectin marks circulating (larvae and adults) and sessile (larvae) hemocytes (30). After 48 hours of feeding, all groups that had DSS included in their treatments showed a higher number of flies with ≥ 20 GFP positive cells in their hindguts compared to those fed on sucrose **(** **Figure 3** Panels I and II). Only 9% of analyzed flies feeding on sucrose had ≥ 20 GFP-positive cells in their hindguts. While the proportion of flies with ≥ 20 GFP-positive cells in their hindguts was 26% in the group treated with DSS for 48 hours, this proportion was 32% in the group treated with DSS and EBV DNA for 48 hours (p= 0.511). On the other hand, 25% of flies in the group of treated with DSS for 24 hours followed by sucrose for 24 hours had ≥ 20 GFP-positive cells in their hindguts was, while this proportion was 55% in the group treated with DSS for 24 hours followed by EBV DNA for 24 hours (p=0.003). Of the DNA-fed groups, this latter group had the highest proportion of flies with ≥ 20 GFP-positive cells in their hindguts. None of the flies that fed on EBV DNA alone for 48 hours showed any GFP-positive cells in their hindguts compared to the control group which was fed on sucrose alone hence showing a significant decrease (p=0.023) compared to the sucrose-fed flies. On the other hand, flies fed on DSS then *S. epidermidis* DNA (bacterial DNA control) or Adenovirus DNA (viral DNA control) did not show a significant increase in hindgut GFP-positive cells compared to the sucrose-fed flies; similar observations were made in flies fed on *S. epidermidis* or Adenovirus DNA without prior treatment with DSS. On the other hand, treatment with bacterial LPS, used as a positive control for inflammation-induction resulted in 86.67% of flies with ≥ 20 GFP-positive cells in their hindguts. By day 8 after ceasing treatments, the number of GFP-positive cells in hindguts of flies fed on DSS or DSS then EBV DNA returned to sucrose-fed fly levels (**Figure 3** Panel III).

**Figure 4:**
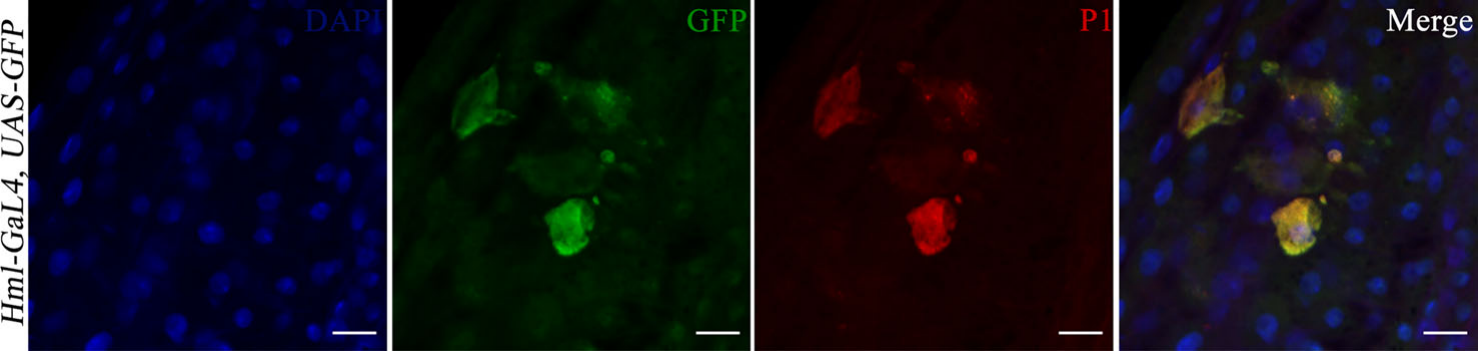
Hindguts of *Hml-Gal4, UAS-GFP* flies after feeding on DSS for 24 hours then EBV DNA for 24 hours. **(A)** DAPI. **(B)** GFP. **(C)** P1. **(D)** Merged DAPI, GFP and P1 images. Scale bar indicates 10 μm.

The 20 GFP-Hemolectin positive cells were selected as a cutoff point since values below 20 were the most recurrently observed in the hindgut of flies feeding on sucrose only for 48 hours. Since a previous report has indicated that the fly gut harbors hemolectin-positive cells that are neuroendocrine rather than hemocytic (31), hindguts were immunostained with a P1 antibody. This antibody recognizes the phagocytic NimC1 receptor expressed on surface of cells with plasmatocytic characteristics (32, 33) (**Figure 4**). Plasmatocytes comprise 95% of the hemocyte population in flies (34). We observed co-localization between GFP-Hemolectin and P1 in the gut of flies feeding on DSS then EBV, hence indicating that these cells are plasmatocytic in nature.

### Circulating Hemocyte Numbers in Adult Flies Fed on DSS then EBV DNA Remain Unchanged

**Figure 5:**
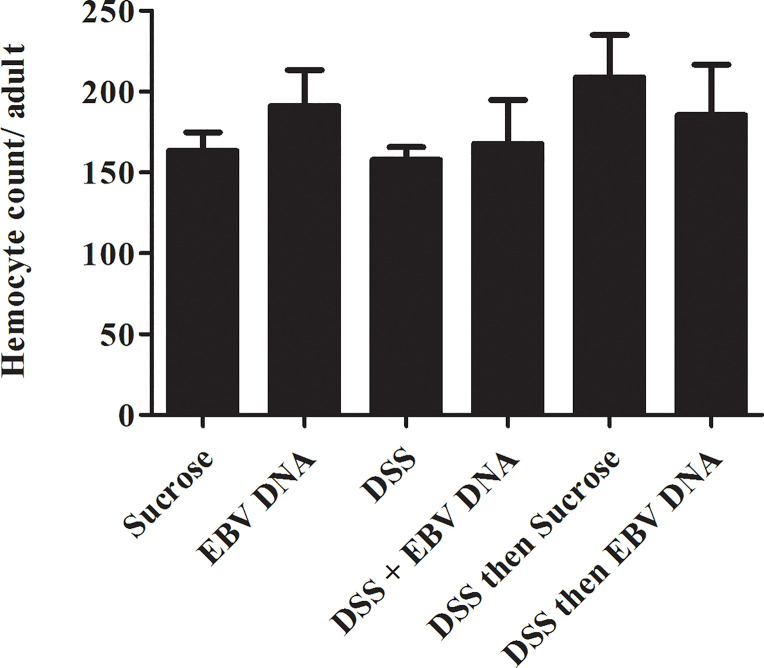
Hemocyte count conducted on *Hml-Gal4, UAS-GFP* hemolymph of adult male flies fed on different regimens of DSS and/or EBV DNA. Sucrose is used as a vehicle in all treatments.

After 48 hours of feeding on the various regimens described above, the number of circulating hemocytes was assessed by collecting and analyzing the adult fly hemolymph. No significant changes were observed between the different groups (**Figure 5**) indicating that the feeding regimens employed did not induce changes at the systemic level within the timeframe assessed.

### Midgut Intestinal Stem Cell Regeneration Is Unaffected by EBV DNA Treatment

**Figure 6:**
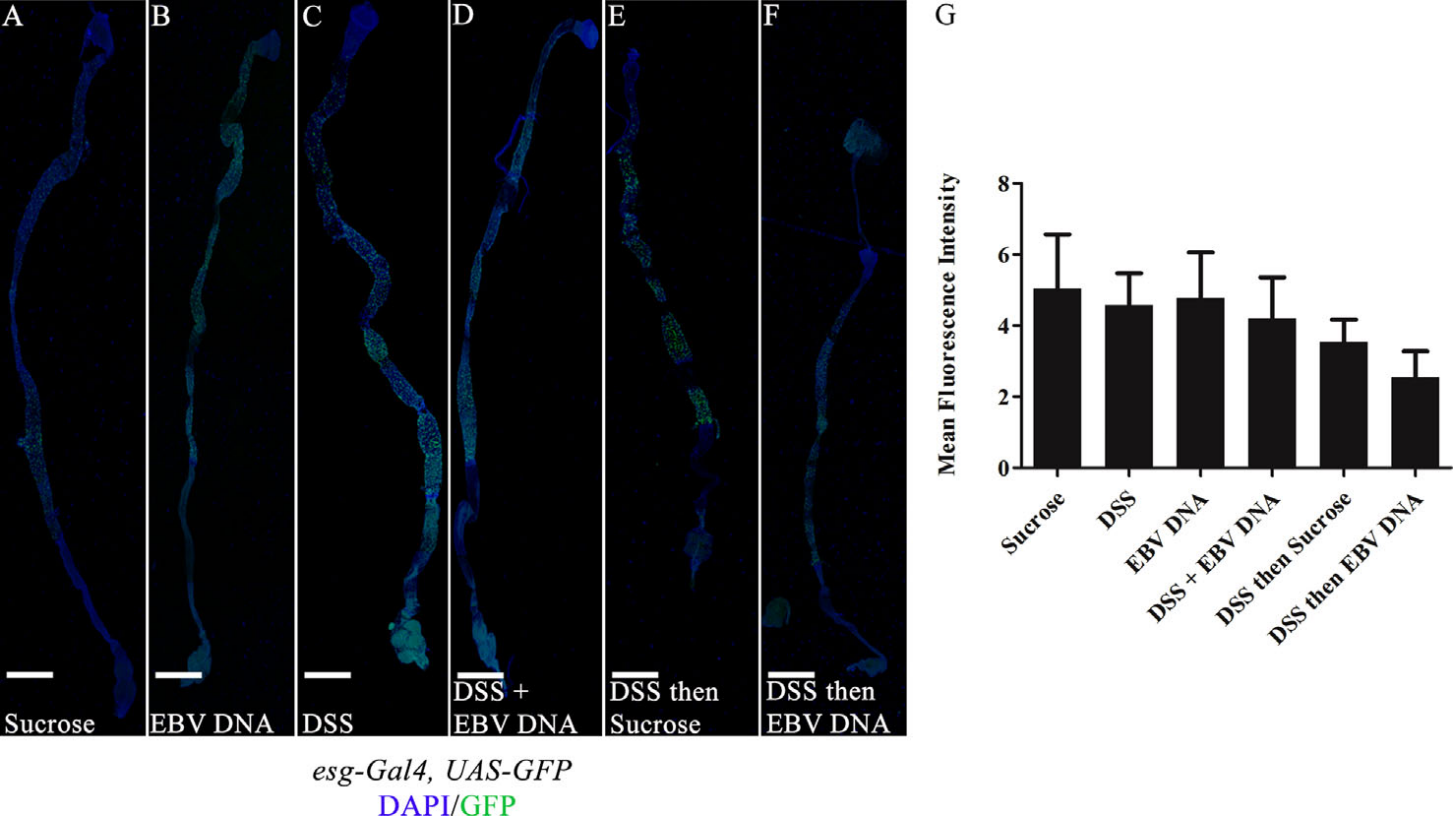
Midguts of *esg-Gal4, UAS-GFP/+* flies fed on different regimens of DSS and/or EBV DNA. **(A)** Flies fed on sucrose (used as vehicle in the other treatments) for 48 hours. **(B)** Flies fed on EBV DNA for 48 hours. **(C)** Flies fed on DSS for 48 hours. **(D)** Flies fed on DSS and EBV DNA for 48 hours. **(E)** Flies fed on DSS for 24 hours then sucrose for 24 hours. **(F)** Flies fed on DSS for 24 hours then EBV DNA for 24 hours. Hemolectin/GFP is in green; DAPI is in blue. Scale bar indicates 400 μm. **(G)** Mean fluorescence intensity of intestinal stem cells (ISCs) and enteroblasts in the midgut of flies after feeding on different regimens of DSS and/or EBV DNA. Sucrose was used as a vehicle.

The fly midgut is known to home ISCs which proliferate upon damage (35). Nevertheless, previous reports have indicated that high molecular weight DSS, such as the one employed in our experiments, does not induce ISC regeneration in the midgut (36). To assess whether inclusion of EBV DNA treatment could exacerbate this, *esg-Gal4, UAS-GFP* flies were used. Escargot (Esg) is a zinc-finger transcription factor expressed in ISCs and enteroblasts of the midgut; this mediator plays a role in maintaining cell identity (35). Examining guts from the different treatments revealed no significant changes in endogenous GFP expression (**Figures 6A–F**). This was further confirmed upon quantifying the mean fluorescence intensity in ISCs in different groups, whereby no significant changes were documented (**Figure 6G**).

### EBV DNA Increases Diptericin and IMD Expression

**Figure 7:**
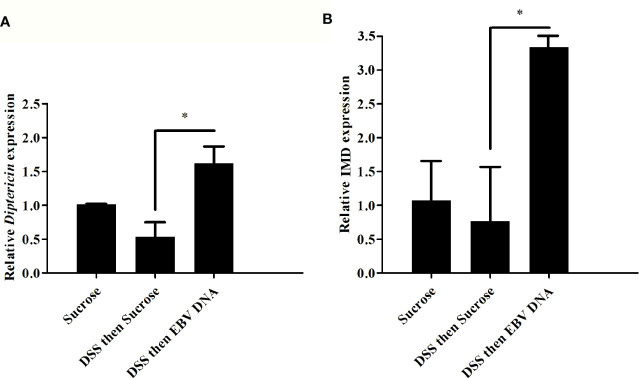
Relative gene expression of **(A)** *Diptericin* and **(B)** IMD in guts from flies fed on different regimens of DSS and/or EBV DNA. Sucrose was used as vehicle. *p-value<0.05.

We previously demonstrated that injecting flies with EBV DNA results in enhanced expression of *Diptericin* as an indicator of the IMD immune pathway activation (9). To assess whether induction of inflammation and EBV DNA treatment would stimulate an innate immune response and specifically alter *Diptericin* expression, the relative gene expression of this mediator was assessed in the guts of adult male flies subjected to the different treatments. A significant 3-fold increase in *Diptericin* expression was observed in the guts of flies fed on DSS then EBV DNA compared to control flies fed on DSS then sucrose (**Figure 7A**). However, no significant increase in *Diptericin* expression levels was observed in the other DNA-fed groups. On the other hand, to examine whether a systemic increase in *Diptericin* expression could be detected, real-time PCR was conducted on RNA extracted from whole flies subjected to the aforementioned treatments; however, no change in the expression was observed (data not shown). Assessing the expression of IMD, which acts upstream of *Diptericin* and is an integral component of the IMD pathway, similarly showed a significant increase of about 4 folds in its expression in the guts of flies fed on DSS then EBV DNA compared to flies fed on DSS then sucrose (**Figure 7B**).

## Discussion

EBV is a prevalent virus that has been associated with different diseases including gastric cancer and IBD. A significant correlation between IBD and gastrointestinal EBV has been reported by multiple groups. Minimal amounts of EBV have been detected in normal gastric tissues but the virus has been highly detectable in gastric lesions from Crohn’s disease and ulcerative colitis tissues. Possible perpetuation of inflammation in ulcerative colitis patients due to the active replication of the virus detected in the inflammatory lesions has been indicated (10). Further research is required to explore the definite role of EBV in IBD. Innate immune responses secondary to EBV DNA administration have previously been shown. Our group previously reported immune stimulation in response to EBV DNA in various models. In mice, pro-inflammatory cytokines were triggered *via* the involvement of TLRs (5–7, 37). In *D. melanogaster*, EBV DNA was capable of triggering the activation of the IMD pathway systemically as well as increasing hemocyte levels in the fly hemolymph (9). In the study at hand, we aimed at assessing the effect of EBV DNA on gut inflammatory responses in *D. melanogaster* by examining markers of the cellular immune response, the humoral IMD response as well as ISC regeneration.

Administration of EBV DNA to flies after inducing gut inflammation resulted in increased levels of hemolectin-positive cells in fly hindguts. Feeding DSS on the first day then EBV DNA on the next showed further enhanced hemolectin-positive cell accumulation in the hindgut in comparison to flies concomitantly fed DSS and EBV DNA. Hence, establishing gut inflammation and then administering EBV DNA likely resulted in a further aggravated cellular response.

Upon assessing the nature of the hemolectin-positive cells we detected in the fly hindgut, we were able to confirm that they are plasmatocytes. While fly hindguts were reported to harbor hemocyte-like cells (38), to the best of our knowledge, this is the first report to indicate that these cells are plasmatocytes. On the other hand, the localization of hemocytes in the midgut region of the gut of *D. melanogaster* has been previously demonstrated (31). Hemocytes were shown to aid in immune responses in the midgut by taking part in the phagocytosis of pathogens and controlling ISC regeneration (31, 39). Hence, possible similar immune contributions may take place in the hindgut of an adult fly upon inducing inflammation.

To assess the effect of EBV DNA on systemic cellular components, hemocytes from the adult fly hemolymph were enumerated after feeding on the various treatments employed in this study for two days. No significant changes in the number of hemocytes were observed; this could be due to a localized damage induced in the gut by DSS and EBV DNA that does not result in systemic changes.

On the other hand, although it has been previously reported that DSS with a high molecular weight, as employed in our studies, does not show an effect on ISC regeneration in the fly midgut, we examined whether inclusion of EBV DNA is capable of triggering regenerational changes (36). Upon assessing the fluorescence intensity of ISCs in the midgut, which is indicative of increased division of stem cells, we did not observe any remarkable indication of regeneration. Therefore, it appears that EBV DNA did not enhance ISC regeneration responses in the midgut; however, it elicited increased counts of hemocytes in the hindgut. This highlights differences in inflammatory responses between the midgut and the hindgut.

Worth noting is that fewer flies with more than 20 hemocytes in fly hindguts were observed in the EBV DNA-fed group and the *S. epidermidis* DNA-fed group compared to the sucrose-fed flies. However, the Adenovirus DNA-fed flies were on par with the controls. These variations may indicate a particular effect of certain nucleic acid molecules at the local level but not at the systemic level. Mechanisms behind these effects and how they pertain to the nature of each type of nucleic acid are worth examining in future studies.

Upon assessing the humoral response in the gut of flies fed on DSS then EBV DNA, we observed that the transcriptional level of *Diptericin* was increased by 3 folds in comparison to flies fed on DSS then sucrose. These results correlate with our previous studies showing the activation of the IMD pathway systemically in response to EBV DNA (9). Studies have shown the role of the IMD pathway in defense against gut bacterial infections (40, 41). Moreover, studies have demonstrated that the IMD pathway is activated in response to accumulation of chromosomal DNA in flies (42). A previous study suggested that the IMD pathway in *D. melanogaster* larvae induces c-Jun N-terminal Kinase (JNK)-dependent PDGFR and VEGFR receptor related (PVR) ligands such as PDGFR and VEGFR receptor related factor 2 (PVF2) and PDGFR and VEGFR receptor related factor 3 (PVF3) which in turn induce the proliferation of hemocytes (43).

It is quite likely that DSS initiates the observed reaction in our study by causing intestinal abrasions that first stimulate the local immune response. Initial responses are expected to be antimicrobial peptide production at the epithelial surface level rather than by hemocytes (44, 45). Once such a response is initiated, it can be exacerbated by the EBV DNA. It seems locally generated signals in this case are not relayed to immune-responsive tissues at the systemic level, such as the fat body; however, to confirm that diptericin in the gut is secreted by gut epithelium in response to DSS damage and then EBV administration further studies are required. The conclusion that some initial damage is required before further immune responses are exacerbated by EBV DNA is supported by our experiments demonstrating that administering DNA, of any type, alone does not induce hemocyte recruitment to the hindgut. This is also similar to what is believed to occur in IBD in humans, whereby some initial tissue damage is exacerbated by microbes, including the normal flora, resulting in inflammation and disease.

In conclusion, our results suggest that oral administration of EBV DNA stimulates the proliferation or accumulation of plasmatocytes and the activation of the IMD pathway in the hindgut of *D. melanogaster*. The IMD pathway is comparable to TNFR signaling in mammals. Hence, assessing whether the TNFR pathway is stimulated by EBV DNA in mammalian gastric tissues can be investigated; such studies may highlight mediators that can serve as potential therapeutic targets to alleviate the inflammatory effects of EBV in IBD.

## Data availability statement

The original contributions presented in the study are included in the article/supplementary material. Further inquiries can be directed to the corresponding authors.

## Author Contributions

JM contributed to the study design, conducted experimental procedures and analysis in addition to writing the first draft of the manuscript. AA, MG, HH, MShe, ZH, and AF contributed to the experimental procedures and data analysis. MShi and ER designed the study, oversaw the experimental work, data analysis and writing. MShi and ER contributed equally to this work. All authors contributed to the article and approved the submitted version.

## Funding

AA is the recipient of National Council for Scientific Research-Lebanon (CNRS-L)/AUB Doctoral Scholarship award. AF is funded by the Medical Practice Plan (MPP) at the American University of Beirut. MShi is funded by the Medical Practice Plan (MPP) at the American University of Beirut and LLS (Leukemia and Lymphoma Society) Canada. ER is funded by the Asmar Research Fund, the Lebanese National Council for Scientific Research (L-CNRS), and by the Medical Practice Plan (MPP) at the American University of Beirut.

## Conflict of Interest

The authors declare that the research was conducted in the absence of any commercial or financial relationships that could be construed as a potential conflict of interest.
